# Experiential student study groups: perspectives on medical education in the post-COVID-19 period

**DOI:** 10.1186/s12909-023-04006-9

**Published:** 2023-01-19

**Authors:** Evgenia Charikleia Lazari, Charalampos C. Mylonas, Georgia Eleni Thomopoulou, Evangelia Manou, Constantinos Nastos, Nikolaos Kavantzas, Emmanouil Pikoulis, Andreas C. Lazaris

**Affiliations:** 1 First Department of Pathology, School of Medicine, National and Kapodistrian University of Athens, 75 Mikras Asias Str., Building 10, GR-115 27 Athens, Greece; 2 Cytopathology Department, “Attikon” University General Hospital, School of Medicine, National and Kapodistrian University of Athens, Athens, Greece; 3 Third Surgical Department, “Attikon” University General Hospital, School of Medicine, The National and Kapodistrian University of Athens, Athens, Greece

**Keywords:** Experiential learning, Interactivity, Interplay, Medical education, Pathology study groups

## Abstract

**Background:**

Undergraduate medical curricula often fail to integrate experiential learning methodologies. Thus, a pilot series of interactive pathology lessons was designed and implemented in an attempt to promote experiential learning.

**Methods:**

Thirty pre-graduate medical students voluntarily participated in the interactive study groups at the First Department of Pathology of the National and Kapodistrian University of Athens, Medical School. A questionnaire was designed to investigate the satisfaction of students regarding their participation in pathology study groups and to identify the characteristics that shape students’ perceptions of the foundations of medical education. Descriptive statistics (mean values) were used to describe the students’ evaluations of the pathology study groups, and thematic analysis was conducted to investigate the data collected using open-ended questions.

**Results:**

Interactions with the professor and the option of co-observing the slides using dual-view optical microscopes and virtual slides were each evaluated as “Excellent” by ≅ 95% of the students. Four overarching themes were identified regarding the core characteristics of medical education according to the students’ perspectives: 1) educational background in medical education, 2) interaction with educators in medical education, 3) educational material in medical education and 4) assessment in medical education.

**Conclusions:**

The high rates of acceptance of the pathology study groups reflect the desire and need for active learning methodologies to be implemented in modern medical education. Nearly all the students mentioned the need for practical skill acquisition, the integration of theory into practice and ethics in medical education. The success of these optional pathology study groups highlights the need for similar modalities to be incorporated into the main medical education curriculum.

## Introduction

Undergraduate medical education curricula in Greece are theory-driven and textbook-based to a considerable extent. They focus on the development of students’ knowledge via traditional lectures and studying textbooks, which, after a period of several years, is to be integrated into clinical practice. Medical professors confront challenges with respect to delivering vast amounts of ever-increasing knowledge effectively, and students often struggle to memorize all this information with only a limited understanding of its clinical significance.

Due to this gap between theory and application, many students and professors have expressed the need to promote experiential learning in the field of medical education. Experiential learning, as its name suggests, refers to the learning that occurs through experience. Based on constructivist adult learning theories and the influential work of John Dewey, experiential learning seems to be a necessity for the formation of a fruitful academic environment for future medical professionals [1, 2].

Moreover, recent medical education literature has highlighted the importance of interaction in learning, both between the learner and the lesson’s content as well as between the learner and the professor. Team-based, interactive lessons are increasing in popularity among health care educators [3–6], as they play a crucial role in facilitating multiple highly effective teaching methodologies, such as problem-based learning [7, 8], case-based learning [9] and peer-assisted learning [10, 11].

Despite evidence indicating the favourable outcomes of the medical teaching strategies mentioned above, Greek undergraduate medical programs often fail to integrate these strategies, mostly due to a large number of students (often exceeding 350 per semester), which restricts the students’ personal engagement and interaction with the professor. Pathology is taught in our department at the 4th and 5th semester of undergraduate studies, without the students having yet started their clinical practice. The educational objectives per chapter are defined in our School’s curriculum (https://school.med.uoa.gr/fileadmin/depts/med.uoa.gr/school/uploads/Odigos_Spoydon/Odigos_Spoydon_2021-2022_web.pdf). All students are classified into 6 practice groups and microscope training can be done through virtual, digitized histological slides. On a weekly basis, the overall training consists of 3 h of theoretical lectures, 1.5 h of macroscopic and microscopic exercises and 1 h of clinicopathological discussion (the latter only in the 5th semester).

University students are generally considered to be the main stakeholders of higher education institutions regardless of the extent to which the latter are research-focused [12]. Student evaluations play a significant role in providing feedback regarding the effectiveness of educators’ teaching and are therefore taken into serious consideration to increase the quality of teaching/learning dynamics [13, 14]. In the context of the COVID-19 pandemic and the associated implementation of e-learning in medical courses [15, 16], major concerns have been raised regarding the participation and engagement of the students in pathology lessons delivered via e-learning. Despite the high levels of flexibility offered by e-learning, whether synchronous or asynchronous, a relatively low participation rate was noticed in our department. In fact, according to previous research conducted in our department during the pandemic, there was a statistically significant drop-out rate of participants during each e-lesson [17]. Τhe reluctance of students to actively participate in distance education led us to evaluate the importance of experiential learning with the physical presence of the teacher, aimed at teacher-student interaction in order that students acquire practical skills. To the best of our knowledge, experiential learning has not so far been adequately reported in the literature, as to be considered a necessary component in medical education curricula.

Thus, after the lifting of measures implemented to combat the pandemic and the return to classroom-based education, a pathology professor working at the School of Medicine at National and Kapodistrian University of Athens (the leading author of this article) took the initiative to design and implement small team-based, interactive pathology study groups that focused on three diagnostic fields (inflammation, neoplasia, and thyroid gland neoplasms); student participation in these groups was optional.

In this study, the authors describe and critically evaluate their attempt to maximize the students’ learning experience via this pilot series of interactive pathology lessons, based on the following research questions: 1. How was our attempt in experiential learning in Pathology evaluated from our students? 2. What characteristics shape the students’ perceptions of the fundamental factors of medical education? Our attempt to implement experiential learning in Pathology teaching in our department stemmed from the belief that our students would positively reinforce our effort, and that they, even without professional training in teaching, are instinctively aware of the founding aspects of education in general.

## Methods

### Structure of the pathology study groups

Of the 256 students enrolled in the pathology course, 30 participated in the pathology study groups consistently. The professor provided the students with up to 12 histological slides per diagnostic field, each of which referred to a different medical case. These slides were also digitized and made readily available via online software. Using the latter, the students could access the digitized slides and study them from their own workplaces and at their own pace. The volunteer participants in the study group were undergraduate medical students and were mostly preclinical. Meetings with the professor were scheduled weekly throughout the spring semester of 2022 and lasted approximately 2 h each.

During the first meeting of each of the three study groups, the selected case studies were presented by the professor with the aid of a compound light optical microscope. Subsequently, the medical students (either as individuals or as small subgroups) were assigned an individual case to study and were provided with the relevant histological slides, both physical and digitized. Their task was to recognize the characteristic histological features of the disease presented in the case and to prepare a brief, relevant PowerPoint presentation about the case. Apart from studying the digitized slides at home, the students were given the opportunity to study the slides collaboratively with their professor using dual-view optical microscopes in the pathology laboratory. Moreover, during the lessons, the professor often encouraged his students to reflect upon the true meaning of medical education in general. Encouraging a holistic approach to pathology, such as ensuring effective communication among the pathologist, the clinician, and other medical specialists (e.g., radiologists) was attempted.

During the final meeting of each study group, all students presented their case studies to their peers with the assistance of the professor. The presentations were broadcast live on YouTube via the Pathology Department’s channel and the “Zoom Meetings” software program so that all 256 students present during the semester could observe them. The presentations were recorded and were subsequently uploaded to the pathology e-class.

### Data collection

To investigate students’ levels of satisfaction with and perceptions of their participation in the interactive pathology study groups, we distributed a questionnaire to all 30 participants via Google Forms. We first issued a short demographic questionnaire and subsequently proceeded to distribute the comprehensive questionnaire. The questionnaire completion time was estimated to be 10 min. To score the students’ responses, we utilized a 10-point Likert scale (0 = totally negative, 10 = totally positive). Moreover, we included open-ended questions to gain valuable insight into the students’ attitudes and suggestions on the topic at hand.

The questionnaire consisted of three parts. The first part included questions aimed at collecting personal information (e.g., the student’s gender, semester of study, occupational status and physical or online participation). The second part concerned the students’ perspectives of their experiences and their evaluations of various aspects of that experience. The third part involved questions that focus on students’ opinions concerning the core characteristics that medical education should generally embrace and suggestions for improving the study groups.

The questionnaire was sent to the students who were enrolled in the pathology study groups following the final team meeting. We collected data between May and June 2022. Of the 30 students who were invited to participate in the groups, 19 responded to our call.

Permission to conduct the study was granted by the ethics committee of our faculty, and all participants provided informed consent prior to participating in the study. The anonymity of the participants was ensured at each stage of the survey.

### Data analysis

We employed both quantitative and qualitative methods to process and analyse the data we collected.

**Fig. 1:**
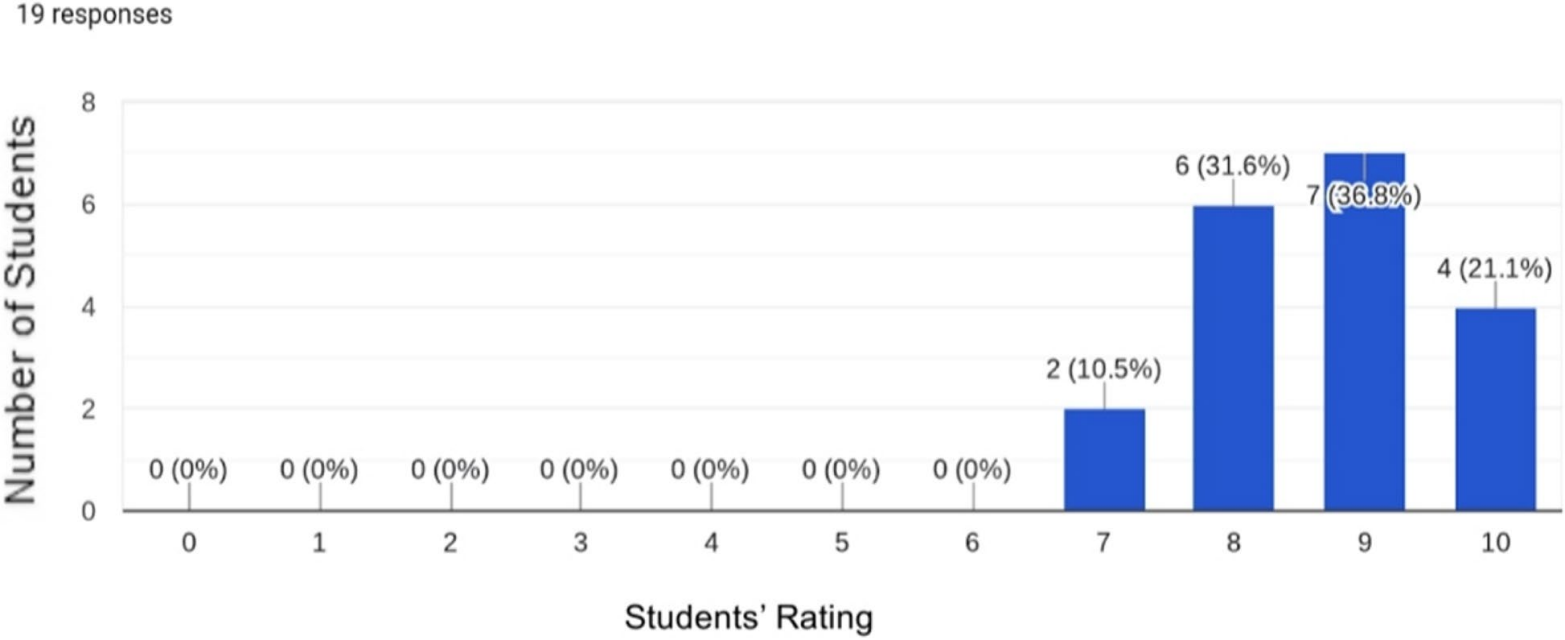
Overall student satisfaction score (in a Likert point scale) concerning the pathology study groups

**Fig. 2:**
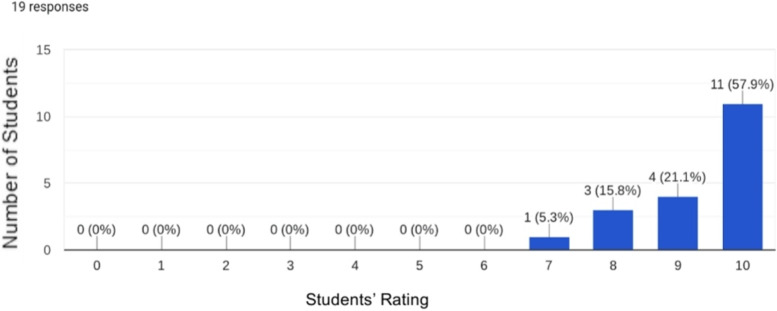
Evaluation of the practical aspect of the group

The answers to the questions scored on the Likert scale were exported to an Excel sheet, which was subsequently used to quantify the number of students who evaluated aspects of the course with the same score. Data were visualized in the form of graphs (Figs. 1 and 2).

The open-ended research questions were analysed using thematic analysis, which aimed to find common themes within the data collected. Thematic analysis is a widely used tool for analysing qualitative data and allows researchers to gain an in-depth understanding of the participants’ perspectives [18].

In accordance with Braun and Clarke’s framework for thematic analysis [19], two of the authors read the students’ answers and made notes to familiarize themselves with aspects of the data that were relevant to our research questions. To avoid subjective bias and improve validity, each of the two authors independently identified broad patterns within the answers, and at regular meetings, they discussed the codes thereby constructed. When all the data were initially coded, the relevant coded data extracts were then organized into four broader themes. This process was subsequently reviewed by all authors, ensuring that it focused on a coherent pattern. The authors subsequently defined and named the four themes thus identified and conducted a more in-depth analysis. Any disagreements in interpretation throughout the process of thematic analysis were resolved by reaching consensus among the authors.

## Results

### Descriptive statistics

In terms of overall satisfaction, the evaluation of the pathology study groups (Fig. 1) featured ratings of “Excellent” (9–10) by nearly 60% of the students who participated and ratings of “Very Good” (7–8) by the rest.

The educational technology tools utilized during the pathology study groups, such as the use of digitized histological slides, were evaluated highly, receiving scores of 9–10 (“Excellent”) from the majority of students (i.e., approximately 95% of the students). The experience of creating PowerPoint presentations regarding the case studies received a slightly lower but still acceptably favourable rating from the majority of the students (80% of whom rated this item as “Excellent”).

Concerning the students’ communication with the professor, it is noteworthy that ≅ 95% of them evaluated their interaction with the professor as “Excellent”, indicating high satisfaction with these meaningful and direct interactions. This claim is also supported by the high evaluation of the offer to co-observe the slides using dual-view optical microscopes (95%).

Moreover, the practical nature of the pathology study groups was widely accepted and received highly evaluations of “Excellent” from approximately 80% of the students. Students’ evaluations of the practical aspects of the pathology study groups are depicted in Fig. 2.

### Thematic analysis

After following Braun and Clarke’s framework for thematic analysis [19] for the initial coding and the subsequent thematic analysis, we identified four overarching themes regarding the core characteristics of medical education according to the students’ perspectives (Table 1).

**Table 1:** Most significant factors in Medical Education

Themes	Definition	Codes
Education Background	Nature of knowledge	• Theoretical • Practical • Integrating theory to practice • Relating Pathology to other Medical Fields • Ethics
Interplay with Educators	Student-Educator relationship	• Guidance • Personal relationship • Encouragement
Educational Material	Learning resources	• Grounded and latest evidence • Asynchronous • Organized • Supplementary • Latest technologies • Time
Assessment	Means of measuring student performance, progress and achievement of learning outcome	• Theory • Practice • Integrating Theory to Practice

### Theme 1: background in medical education

The elements of the educational background that were considered important for medical education were a) theoretical knowledge, b) practical knowledge, c) the integration of theory into practice, d) the relation between pathology and other medical fields and e) ethics in education. The pathology study group in which the students participated endorsed all those elements, with a special focus on elements c, d, and e. The students learned about inflammation, neoplasia, and thyroid gland neoplasms not merely by studying textbooks but also through the purposeful and self-immersive tasks associated with the study group. Moreover, ethics in education was promoted through the discussions concerning educational philosophy orchestrated by the professor.

It is noteworthy that the majority of the students highlighted the importance of practical skills, the integration of theory into practice and ethics. Nearly all the students reported the need for practical skill acquisition and the integration of theory into practice. Specifically, four students highlighted their need for practical knowledge through their preclinical classes. As another student stated,

“The implementation of theoretical knowledge in practice is vital for the knowledge to be preserved long-term and not get lost in oblivion.”

Interestingly, half of the students mentioned ethics as a significant factor in medical education, while four of them listed “empathy”, “a humanistic approach”, “a moral and inclusive medical education” and the “development of critical thinking in contrast to rote learning” as the most important factors.

### Theme 2: interaction with educators in medical education

Another factor that emerged as crucial in medical education pertained to the relationships between the students and their educators. An ideal relationship with the educators consisted of 1) guidance in the students’ search for knowledge, 2) positive reinforcement from the educators and 3) meaningful, personal engagement in the subject being taught.

The majority of the students included interaction with educators as a major aspect of knowledge acquisition. In fact, 20% of them regarded their personal relationship with the professor as the most important component of medical education. The role of the educator was considered by one of the respondents to be “a guide to exploring the massive amounts of knowledge” and by another to be oriented towards “the meaningful communication of knowledge, avoiding overwhelming information overload”. Most students expressed a desire for a positive, personal relationship with their educators, who would inspire them with their passion and encourage them in their own search for knowledge. The need for respectful understanding of the students’ needs in terms of their mental health and their demanding schedules were also mentioned by two of the students in the context of ways of enhancing student-educator collaboration.

### Theme 3: materials in medical education

Regarding the ideal material for medical education, the students mentioned a broad set of traits. As far as technological factors are concerned, some students regarded the utilization of the latest technologies in the lessons as important, including, more specifically, opportunities for high quality asynchronous e-learning. Three students mentioned the value of the comprehensive organization of the educational material, while two of them expressed the need for free access to the latest evidence-based textbooks. Some students noted that optional, additional material would enhance their educational experience.

Another important factor identified in the context of medical education was time. Students stated that the lessons should allow the students to have the necessary time to familiarize themselves with and consolidate the information involved in medical education.

### Theme 4: assessment in medical education

The final theme that emerged was the significance of assessment in medical education. One student noted that it is important for educators to assess their students' abilities to apply theory in practice. As this student mentioned,

"The grades shouldn't only reflect theoretical knowledge but also the student's ability to integrate theory into practice and perform practical tasks."

Another student highlighted the importance of university exams that focus on the essence of each subject and the students’ genuine understanding thereof.

## Discussion

The purpose of the interactive pathology study groups was not to cover the entire pathology curriculum. Rather, the focus was to promote experiential learning on this subject by encouraging the students’ active participation and contributing to their learning process practically.

Experiential learning is defined as a constructivist approach to learning through which the students create their personal meaning from experience in accordance with their individual learning styles by following Kolb’s learning cycle [20–22]. Kolb’s learning cycle entails the following steps: concrete experience, reflective observation, abstract conceptualization and active experimentation [20, 21]. The opportunity to apply one’s acquired knowledge in practice and the promotion of learning via reflection are only some of the benefits of experiential learning with respect to deepening and strengthening the learning process. The importance of applying this concept to undergraduate medical students is apparently related to their future training as medical professionals. This educational model could also be implemented to other age-groups in the context of life-long learning.

An interesting descriptive finding of this study was the high evaluations of co-observing the slides through dual-view optical microscopes (an option that was rated by 95% of the students as “Excellent”). This aspect of the pathology study groups aimed to promote direct, personal relationships with the educator. As mentioned previously, the interaction between the students and their professor was the most highly evaluated item included in the questionnaire. Regarding the thematic analysis, the majority of students identified interacting with the educator as a major aspect associated with their knowledge acquisition, and 20% of them regarded their personal relationships with the professor as the most important component of their medical education.

Student–educator interaction has been studied in depth in medical education research. According to Simpson and Galbo [23], the art of teaching is fundamentally rooted in student-professor interaction. Clinical skills training [24] and identity formation are both strongly influenced by the interpersonal student-educator relationship. Additionally, fostering interpersonal relationships between the educator and his or her students leads to the development of skill expertise or changes in perspectives and motivation [25]. The existence of personal positive communication between professor and student cultivates the appropriate communication skills of the student by stimulating his or her emotional intelligence; the latter, it is known, is crucially related to his or her future professional success.

The practical nature of the interactive pathology study groups was also widely accepted and evaluated as Excellent by approximately 80% of the students. Nearly all the students reported the need for practical skill acquisition, the integration of theory into practice and ethics in medical education. It is noteworthy that, much to the authors’ surprise, most medical students were not familiar with adult learning theories and teaching methodologies but nevertheless intrinsically recognized the significance of all these factors in learning.

Apart from finding ways of effectively transmitting vast amounts of medical knowledge, the modern medical community is also concerned about the “dehumanization” of medical practice [26, 27] and the loss of compassion and empathy in the training of students [28–30]. Medical education often fails to promote students' orientation towards empathy, leading to a phenomenon called “ethical erosion” among medical students during their transition from preclinical to clinical training and beyond, in their transition to a specialization/residency and independent practice [31–33].

High-quality student–teacher relationships are associated with students' intrinsic motivation to learn [34, 35] and have a major impact on learning as well as on students' sense of social identity. This “identity-forming” aspect of the student–teacher interpersonal relationship can shape students' professional choices and behaviours and can be harnessed by a teacher who focuses on promoting empathy when teaching his or her class. By cultivating personal, human communication between instructor and medical student, the future doctor encounters valuable stimuli, which ultimately enable him or her to cultivate empathy towards his or her patients. Educators should serve as role models [36] for their students, guiding and inspiring them.

Medical education at all levels should be meticulously designed to equip students with the knowledge, clinical skills, and professionalism that are required to become competent physicians. In the Greek reality of the last 40 years, higher education, at least in Medicine, has been characterized by generally impersonal professor-student relationships and by a one-sided strategy on students’ part, not aimed at the acquisition of knowledge, but solely at the success in the exams of each course. The above problematic view of education intensified during the pandemic. In fact, concerning the regulations of higher educational institutes in Greece, one article published in Government Gazette [37] explicitly states the following: “Educators who insist on traditional teaching methodologies should immediately alter those practices. This particular change of approach to the core of education aims at the transition from solely teaching into efficiently promoting learning. The educators’ responsibility isn’t merely the delivery of information but also teaching students how to learn. Even humanitarian studies, such as Law, should incorporate practical lessons into their curricula". Despite the 40 years that have passed since the publication of this article, a long road lies ahead towards the implementation of this approach in higher educational institutes.

The high rate of acceptance of the interactive pathology study groups and the overall satisfaction exhibited by the students who participated in them in our study reflect the desire and need for active learning methodologies in modern medical education. The success of these optional pathology study groups highlights the need for similar modalities to be incorporated into the main medical education curriculum. The findings of this research deserve consideration by the education committee of our Medical School in order that a new teaching practice is shaped by the educators.

### Limitations

One limitation that has to be taken into account in our study is the fact that 11 students of those who participated in our study groups did not answer the evaluation questionnaire. This could be related to them not being satisfied with our attempt, or it could be due to other, random factors. Meticulous efforts are needed in future related research to minimise the missing data, and, therefore, ensure the representativeness of our sample.

## Data Availability

The datasets analyzed during the current study are available from the corresponding author on reasonable request.
